# Scalable non-negative matrix tri-factorization

**DOI:** 10.1186/s13040-017-0160-6

**Published:** 2017-12-29

**Authors:** Andrej Čopar, Marinka žitnik, Blaž Zupan

**Affiliations:** 1 Faculty of Computer and Information Science, University of Ljubljana, Ljubljana, Slovenia; 2 Department of Computer Science, Stanford University, Stanford, 94305 CA USA; 3 Baylor College of Medicine, Houston, 77030 TX USA

**Keywords:** Matrix factorization, Non-negative matrix tri-factorization, Non-negative block value decomposition, Block-wise multiplication, Graphics-processing unit, Large scale latent factor analysis

## Abstract

**Background:**

Matrix factorization is a well established pattern discovery tool that has seen numerous applications in biomedical data analytics, such as gene expression co-clustering, patient stratification, and gene-disease association mining. Matrix factorization learns a latent data model that takes a data matrix and transforms it into a latent feature space enabling generalization, noise removal and feature discovery. However, factorization algorithms are numerically intensive, and hence there is a pressing challenge to scale current algorithms to work with large datasets. Our focus in this paper is matrix tri-factorization, a popular method that is not limited by the assumption of standard matrix factorization about data residing in one latent space. Matrix tri-factorization solves this by inferring a separate latent space for each dimension in a data matrix, and a latent mapping of interactions between the inferred spaces, making the approach particularly suitable for biomedical data mining.

**Results:**

We developed a block-wise approach for latent factor learning in matrix tri-factorization. The approach partitions a data matrix into disjoint submatrices that are treated independently and fed into a parallel factorization system. An appealing property of the proposed approach is its mathematical equivalence with serial matrix tri-factorization. In a study on large biomedical datasets we show that our approach scales well on multi-processor and multi-GPU architectures. On a four-GPU system we demonstrate that our approach can be more than 100-times faster than its single-processor counterpart.

**Conclusions:**

A general approach for scaling non-negative matrix tri-factorization is proposed. The approach is especially useful parallel matrix factorization implemented in a multi-GPU environment. We expect the new approach will be useful in emerging procedures for latent factor analysis, notably for data integration, where many large data matrices need to be collectively factorized.

**Electronic supplementary material:**

The online version of this article (doi:10.1186/s13040-017-0160-6) contains supplementary material, which is available to authorized users.

## Background

Biomedical data are becoming increasingly challenging to analyze due to their sheer volume and complexity. Dimensionality reduction approaches address challenges in modern biomedical data analytics by learning useful projections of data into a smaller, compact and pattern-rich latent space. An especially popular dimensionality reduction approach uses matrix factorization [1]. Numerous non-negative matrix factorization methods have successfully been used for gene expression analysis [2–4], patient stratification [5], drug-target interaction discovery [6], gene phenotyping [7], and magnetic resonance image analysis [8–10].

Non-negative (two-factor) matrix factorization considers as input a data matrix **X** and learns two latent factors, **U** and **V**, such that their product **U** **V** approximates **X**, **X**≈**U** **V**, under some criterion of approximation error. One class of non-negative matrix factorization approaches is non-negative matrix tri-factorization, which extends a two-factor model by introducing a third latent factor **S**, such that **X**≈**U** **S** **V** ^*T*^ [11]. This representation is more appropriate for non-square data because it explicitly models data interactions through a latent factor **S** [12]. Several optimization techniques for parallel non-negative (two-factor) matrix factorization have recently been proposed [13–15]. These techniques first partition matrix **X** into blocks and then exploit the block-matrix multiplication when learning **U** and **V**. However, such a straightforward approach does not apply to matrix tri-factorization because, as we show in the Methods section, the learning of any block of **U** and **V** depends on factor **S**.

In this paper, we develop a principled mathematical approach and an algorithmic solution to latent factor learning for non-negative matrix tri-factorization. While there exists an initial solution to speed up the latent factor learning procedure using accelerated matrix operations on a MapReduce cluster [16], this approach is not optimal because it requires a specialized architecture [17]. Even more importantly, in the case of two-factor non-negative matrix factorization, it was shown that the MapReduce-based approach was outperformed by block-wise approaches by two orders of magnitude [18]. Block-wise approaches also provide the means for load balancing. These related studies thus encourage the development of a block-wise approach for matrix tri-factorization.

The paper makes the following contributions. We develop a block-wise approach for matrix tri-factorization. The new approach enables fast factorization on concurrent systems, such as multi-processor and multi-GPU architectures. We report on two variants of the approach: one variant for orthogonal matrix factorization [11] and the other for non-orthogonal matrix factorization [19]. We provide implementation of the new approach for both multi-processor and multi-GPU architectures. We evaluate the proposed approach with respect to dataset shape and size, parallelization degree, factorization rank, and data sparsity. In experiments on several biomedical datasets, we demonstrate that the new approach provides substantial speedups. The speedup is most pronounced on multi-GPU architectures, where matrix tri-factorization can be more than 100-times faster than its serial counterpart.

## Methods

We start by describing the notation, factorization model, and matrix tri-factorization algorithm. The algorithm starts by initializing the latent factors, which are then iteratively revised until convergence. We then introduce a block data representation and provide an algorithm for partitioning data matrices into blocks. Finally, we develop the block-wise latent factor update rules and present the block-wise matrix tri-factorization algorithm.

### Preliminaries: non-negative matrix tri-factorization

Consider a non-negative data matrix ${\mathbf {X}} \in \mathbb {R}^{n \times m}_{+}$, where *n* rows typically describe data instances and *m* columns provide their features. Non-negative matrix tri-factorization (NMTF) learns a decomposition of **X** into three latent factors $\mathbf {U} \in \mathbb {R}_{+}^{n \times k_{1}}$, $\mathbf {S} \in \mathbb {R}_{+}^{k_{1} \times k_{2}}$, and $\mathbf {V} \in \mathbb {R}_{+}^{m \times k_{2}}$ by minimizing the reconstruction error $F({\mathbf U},{\mathbf S},{\mathbf V})=\left \|{\mathbf X}-{\mathbf U}{\mathbf S}{\mathbf V}^{T}\right \|_{Fro}^{2}$ [20, 21]. Columns in factors **U** and **V** are latent vectors and provide the basis of the vector space into which the data (columns and rows of **X**, respectively) are projected. Factorization ranks *k*_1_,*k*_2_≪ min(*m*,*n*) are model parameters that specify the number of latent vectors.

Reconstruction error is typically minimized using the multiplicative update rules [1]. The rules are derived by computing the gradient of the reconstruction error *F* with respect to model parameters **U**, **S**, and **V** and by solving the gradient equations for the model parameters. This procedure results in the following set of update rules [19]:

1 $$\begin{array}{@{}rcl@{}} \mathbf{U} \leftarrow \mathbf{U} \circ \frac{\mathbf{X} \mathbf{V} \mathbf{S}^{T} }{ \mathbf{U} \mathbf{S} \mathbf{V}^{T} \mathbf{V} \mathbf{S}^{T}}  \end{array} $$

2 $$\begin{array}{@{}rcl@{}} \mathbf{V} \leftarrow \mathbf{V} \circ \frac{\mathbf{X}^{T} \mathbf{U} \mathbf{S} }{ \mathbf{V} \mathbf{S}^{T} \mathbf{U}^{T} \mathbf{U} \mathbf{S}}  \end{array} $$

3 $$\begin{array}{@{}rcl@{}} \mathbf{S} \leftarrow \mathbf{S} \circ \frac{\mathbf{U}^{T} \mathbf{X} \mathbf{V} }{ \mathbf{U}^{T} \mathbf{U} \mathbf{S} \mathbf{V}^{T} \mathbf{V}}  \end{array} $$

where ∘ represents the element-wise product and the division is performed element-wise. The matrix tri-factorization algorithm starts by initializing latent factors using small random values and then iteratively applies the update rules in Eqs. (1–3) until convergence [1].

An often-desired variant [11] of matrix tri-factorization imposes orthogonality constraints on the latent vectors. Orthogonality helps in data interpretation because latent vectors are independent of each other and can thus be associated with a particular combination of input features (for **U**) or input data instances (for **V**) [22, 23]. The objective function of orthogonal matrix tri-factorization is $F({\mathbf U},{\mathbf S},{\mathbf V})=\left \|{\mathbf X}-{\mathbf U}{\mathbf S}{\mathbf V}^{T}\right \|_{Fro}^{2}$, under the constraint that **U** ^*T*^ **U**=**I** and **V** ^*T*^ **V**=**I**, where **I** is an identity matrix. Following a similar procedure of gradient computation as described above, we arrive at the following update rules for orthogonal matrix tri-factorization [11]:

4 $$\begin{array}{@{}rcl@{}} \mathbf{U} \leftarrow \mathbf{U} \circ \sqrt{\frac{\mathbf{X} \mathbf{V} \mathbf{S}^{T} }{ \mathbf{U} \mathbf{U}^{T} \mathbf{X} \mathbf{V} \mathbf{S}^{T}} }  \end{array} $$

5 $$\begin{array}{@{}rcl@{}} \mathbf{V} \leftarrow \mathbf{V} \circ \sqrt{\frac{\mathbf{X}^{T} \mathbf{U} \mathbf{S} }{ \mathbf{V} \mathbf{V}^{T} \mathbf{X}^{T} \mathbf{U} \mathbf{S}} }  \end{array} $$

6 $$\begin{array}{@{}rcl@{}} \mathbf{S} \leftarrow \mathbf{S} \circ \sqrt{\frac{\mathbf{U}^{T} \mathbf{X} \mathbf{V} }{ \mathbf{U}^{T} \mathbf{U} \mathbf{S} \mathbf{V}^{T} \mathbf{V}} }  \end{array} $$

### Block-wise multiplicative update rules

**Fig. 1:**
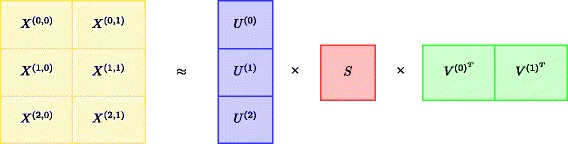
Bock-wise partitioning of data matrix **X** using a 3×2 configuration. Latent factor **U** is divided into three blocks, **U** ^(*i*)^, and latent factor **V** ^(*j*)^ is divided into two blocks. The remaining latent factor **S** is not partitioned into blocks

We present a block-wise formulation of multiplicative update rules for NMTF. We partition the input data **X** into *N*×*M* blocks, **X** ^(*i*,*j*)^, where *i*∈{0,1,…,*N*−1} and *j*∈{0,1,…,*M*−1}. Conversely, latent factor **U** is row-partitioned into *N* blocks, and **V** is column-partitioned into *M* blocks. Figure 1 shows an example where matrix **X** is row-partitioned into *N*=3 blocks and column-partitioned into *M*=2 blocks.

Using this block-wise data representation we reformulate the multiplicative update rules from Eqs. (1–3) as follows:

7 $$\begin{array}{*{20}l} \mathbf{U}^{(i)} & \leftarrow \mathbf{U}^{(i)} \circ \frac{\sum_{j} \mathbf{X}^{(i,j)} \left(\mathbf{V}^{(j)} \mathbf{S}^{T}\right) }{ \mathbf{U}^{(i)} \sum_{j} \left(\mathbf{S} \left(\left(\mathbf{V}^{(j)}\right)^{T}\right) \left(\mathbf{V}^{(j)} \mathbf{S}^{T}\right)\right)}  \end{array} $$

8 $$\begin{array}{*{20}l} \mathbf{V}^{(j)} & \leftarrow \mathbf{V}^{(j)} \circ \frac{\sum_{i} \left(\left(\mathbf{X}^{(i,j)}\right)^{T} \mathbf{U}^{(i)}\right) \mathbf{S} }{ \mathbf{V}^{(j)} \mathbf{S}^{T} \sum_{i} \left(\left(\mathbf{U}^{(i)}\right)^{T} \mathbf{U}^{(i)}\right) \mathbf{S}}  \end{array} $$

9 $$\begin{array}{*{20}l} \mathbf{S} & \leftarrow \mathbf{S} \circ \frac{\sum_{j} \sum_{i} \left(\left(\mathbf{U}^{(i)}\right)^{T} \mathbf{X}^{(i,j)}\right) \mathbf{V}^{(j)} }{ \sum_{i} \left(\left(\mathbf{U}^{(i)}\right)^{T} \mathbf{U}^{(i)}\right) \mathbf{S} \sum_{j}\left(\left(\mathbf{V}^{(j)}\right)^{T} \mathbf{V}^{(j)}\right)}  \end{array} $$

where *i* and *j* denote *i*-th row and *j*-th column matrix block, respectively. Notice that our block partitioning scheme and update rules in Eqs. (7–9) preserve all properties of factorizing a non-partitioned matrix **X**. That is, the result of block-wise matrix tri-factorization is identical to the result returned by non-partitioned matrix tri-factorization as proposed by Long et al. [19]. For example, consider an update for factor **U** in Eq. (1) and its block-wise variant in Eq. (7). To show that these two update rules are equivalent, we need to check that the values in **U** ^(*i*)^ are identical to the values of **U** at corresponding positions. Notice that division in both updates is element-wise; hence, we can independently check equivalency of numerator and denominator. For example, the numerator in Eq. (1) is expressed as **X** **V** **S** ^*T*^. An *i*-th row of this expression can be written in a block-wise manner as $\sum _{j}{\mathbf X^{(i,j)}}{\mathbf V^{(j)}}{\mathbf S}^{T}$, which is exactly the corresponding numerator in Eq. (7). The equivalency of other terms of non-partitioned and block-wise updated rules are further shown in the proof of equivalence of block-wise and non-block-wise formulation of NMTF (Additional file 1: Section 1).

Next, we propose update rules for block-wise orthogonal matrix tri-factorization:

10 $$\begin{array}{*{20}l} \mathbf{U}^{(i)} & \leftarrow \mathbf{U}^{(i)} \circ \sqrt{\frac{\sum_{j} \mathbf{X}^{(i,j)} \left(\mathbf{V}^{(j)} \mathbf{S}^{T}\right) }{ \mathbf{U}^{(i)} \sum_{i} \left(\mathbf{U}^{(i)}\right)^{T} \sum_{j} \left(\mathbf{X}^{(i,j)} \left(\mathbf{V}^{(j)} \mathbf{S}^{T}\right)\right)} }  \end{array} $$

11 $$\begin{array}{*{20}l} \mathbf{V}^{(j)} & \leftarrow \mathbf{V}^{(j)} \circ \sqrt{\frac{\sum_{j} \left(\left(\mathbf{X}^{(i,j)}\right)^{T} \mathbf{U}^{(i)}\right) \mathbf{S} }{ \mathbf{V}^{(j)} \sum_{j} \left(\mathbf{V}^{(j)}\right)^{T} \sum_{i} \left(\left(\mathbf{X}^{(i,j)}\right)^{T} \mathbf{U}^{(i)}\right) \mathbf{S}} }  \end{array} $$

12 $$\begin{array}{*{20}l} \mathbf{S} & \leftarrow \mathbf{S} \circ \sqrt{\frac{\sum_{j} \sum_{i} \left(\left(\mathbf{U}^{(i)}\right)^{T} \mathbf{X}^{(i,j)}\right) \mathbf{V}^{(j)} }{ \sum_{i} \left(\left(\mathbf{U}^{(i)}\right)^{T} \mathbf{U}^{(i)}\right) \mathbf{S} \sum_{j} \left(\left(\mathbf{V}^{(j)}\right)^{T} \mathbf{V}^{(j)}\right)} }  \end{array} $$

The formulation is identical to the non-block-wise formulation originally proposed in Ding et al. [11] and shown in Eqs. (4–6). As before, this property is important because it indicates the proposed block-wise update rules yield latent factors that are identical to the non-block-wise update rules in Ding et al. [11].

### Matrix partitioning

To effectively partition data matrix **X** and latent factors **U**, **V** and **S** into blocks, we distinguish between sparse and dense data matrices. In general, most elements of sparse data matrices are zero, whereas most elements of dense matrices are nonzero [24]. In the case of dense matrix **X**, our matrix partitioning procedure splits **X** into contiguous blocks of approximately equal size. In the case of sparse matrix **X**, we adapt the block size such that each block contains approximately equal number of nonzero elements. Such partitioning leads to workload balancing when factorization is carried out in parallel.

**Fig. 2:**
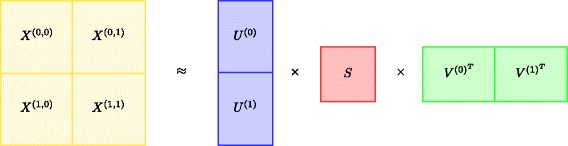
Block-wise partitioning of data matrix **X** using a 2×2 configuration. Latent factors **U** and **V** are each partitioned into two blocks. The remaining latent factor **S** is not partitioned into blocks



The details of matrix partitioning are provided in Algorithm 1. The algorithm takes as input a data matrix **X** and a desired block-wise configuration and returns an appropriate partitioning of **X**. Additional parameters are the number of row blocks *N* and column blocks *M*. Partitioning of latent factors **U**, **V** and **S** is determined by the partitioning of matrix **X** (for example, see Fig. 2).

### Overview of block-wise matrix tri-factorization

A complete algorithm for matrix tri-factorization is given as Algorithm 2. The algorithm starts with matrix partitioning, followed by initialization of latent factors. Initial latent factors are then iteratively refined until convergence using the proposed block-wise multiplicative update rules. While not the subject of this paper, convergence is heuristically determined by observing the value of the objective function or the quality of latent factors and corresponding reconstruction error [11, 19].



## Data and experimental setup

To test the benefits of the block-wise tri-factorization approach, we implemented the approach on multi-processor and multi-GPU architecture. We then tested the implementation on several biomedical datasets. Here, we describe the datasets, evaluation approach and implementation details.

### Data

We considered the following six datasets (Table 1):

- *TCGA-BRCA* is an RNA-Seq gene expression dataset from the GDC databases [25]. The dataset contains expression measurements [26] of genes and gene variants from almost 1,300 human samples.

**Table 1:** Summary of datasets

Dataset	Database	Rows	Columns	Shape	Data type	Density	Nonzero
Fetus	GIANT [29]	25,569	25,608	rectangular	sparse	4.7%	31M
TCGA-BRCA	GDC [25]	1,222	60,483	wide	dense	100.0%	74M
E-TABM-185	ArrayExpress [28]	5,896	22,283	tall	dense	100.0%	131M
Retina	GIANT [29]	25,823	25,822	rectangular	dense	22.0%	147M
Cochlea	GIANT [29]	25,824	25,824	rectangular	dense	42.0%	280M
TCGA-Methyl	GDC [25]	10,181	485,577	wide	dense	81.4%	3841M

We manually categorized each data matrix into three shapes: tall datasets have substantially more rows than columns, wide datasets vice versa, and rectangular datasets have a comparable number of rows and columns. Density denotes the fraction of nonzero matrix elements. The number of nonzero elements in each matrix is given in the last column
- *E-TABM-185* is a microarray gene expression dataset [27] available at ArrayExpress database with accession number E-TABM-185 [28]. It contains gene expression measurements from almost 6,000 human samples representing different cell and tissue types.
- *Fetus* denotes the fetus-specific functional interaction network from the GIANT database [29]. This is a network on human genes where two genes are connected if they are specifically co-expressed in fetal tissue. The fetus-specific gene interaction network [30] has 30 million interactions and is the sparsest network dataset in the GIANT database.
- *Retina* denotes the retina-specific functional interaction network from the GIANT database [29]. This is a network on human genes where two genes are connected if they are specifically co-expressed in retinal tissue. The retina-specific gene interaction network [31] has 147 million interactions.
- *Cochlea* denotes the cochlea-specific functional interaction network from the GIANT database [29]. This is a network on human genes where two genes are connected if they are specifically co-expressed in cochlear tissue. The cochlea-specific gene interaction network [32] has 280 million interactions and is the densest network dataset in the GIANT database.
- *TCGA-Methyl* is a DNA methylation dataset from the GDC database [25], which contains 10,181 samples from Illumina Human Methylation 450 platform [33]. Each sample contains methylation beta values for over 485,577 CpG sites.

### Experimental setup

We factorized each dataset on multi-processor and on multi-GPU architectures. To asses the runtime statistics for a single iteration of factorization, factorization was run for 100 iterations, and measurements were averaged across ten runs. To test relationship between scalability and factorization rank, we varied parameter *k*, such that *k*∈{10,20,…,100}. For a given dataset and a given value of factorization rank, factor matrices were initialized to the same values across different platforms.

We considered the following runtime metrics:

- **Speedup** was expressed as the ratio between processing time *t*_*A*_ for single iteration on observed architecture *A*, and processing time *t*_*CPU*−1_ on a single-CPU: $s_{\mathrm A} = \frac {t_{A}}{t_{CPU-1}}$.
- **Efficiency** was expressed as a fraction of linear speedup, a speedup that assumes that *p* processing units would reduce the runtime by a factor of *p*. For an system *A*_*p*_ with *p* processing units, we compute efficiency relative to performance of a single-unit system *A*_1_ on the same architecture. The formula is: $E_{A_{p}} = \frac {T_{A_{1}}}{p T_{A_{p}}}$. Efficiency of $E_{A_{p}}=1$ indicates a linear speedup. Communication cost pushes efficiency below this optimal value.

### Implementation

We implemented the block-wise matrix factorization in a Python module. To support multi-GPU architecture we use PyCUDA [34]. Communication between processing units uses OpenMPI [35] with Mpi4py Python interface [36]. Matrix operations are accelerated with OpenBLAS [37] on multi-processor architectures and CuBLAS [38] on GPUs. On multi-processor architectures we use NumPy for dense matrices and SciPy for operations on sparse matrices. On GPUs we use Scikit-cuda [39] for dense matrix operations and CuSPARSE [40] with Python-cuda-cffi [41] for operations on sparse matrices. Our implementation is available online [42].

**Fig. 3:**
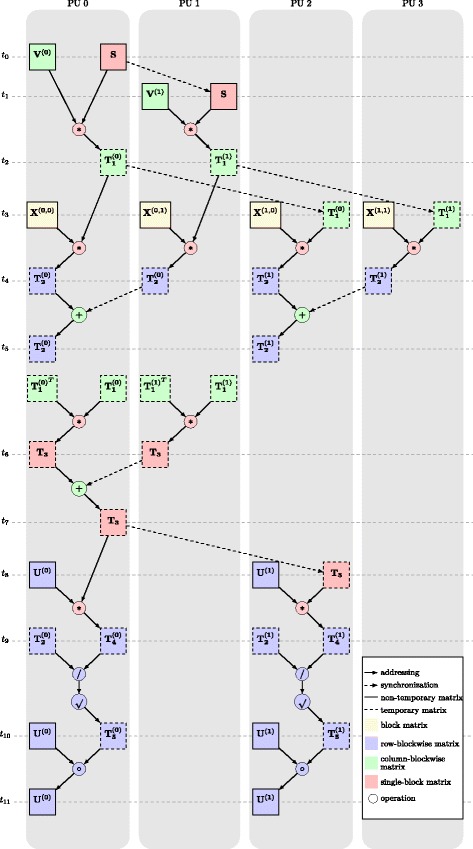
Computational and data transfer workflow for block-wise update of factor matrix **U** on architecture with four processing units and data matrix **X** partitioned into 2×2 blocks. Each vertical band represents a processing unit (PU0 to PU3). Stages where all data are available for the next wave of asynchronous operations are horizontally aligned and are marked with *t*_*i*_, *i*∈{0,1,…,11}

All experiments were run on a computational server with Intel Xeon E5-1650 processor and on four NVIDIA Titan X (Maxwell) GPUs, each with 12 GB of memory. Given *p* processing units, we split input data matrix **X** into *p* blocks, testing various block configurations. Each block was passed to a processing unit that communicated the block with other units when data for next computational steps were required. Figure 3 shows an example of this computational and data transfer workflow for one update of matrix **U** on a 2×2-block configuration. Notice this workflow applies to both 4-GPU and 4-processor architecture.

## Results

We here present results for non-orthogonal block-wise matrix tri-factorization. Results for orthogonal block-wise matrix tri-factorization are qualitatively the same and are provided in the Additional file 1.

**Fig. 4:**
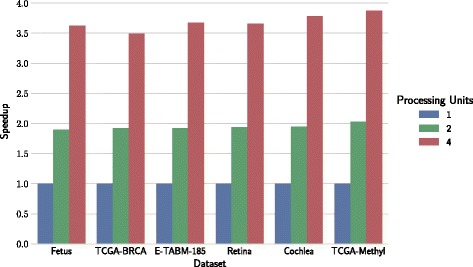
Computational speedups on multi-processing architectures. Speedup using 1, 2 and 4 processes compared to a configuration with one processor. Datasets are ordered from smallest to largest based on the number of non-zero values

**Fig. 5:**
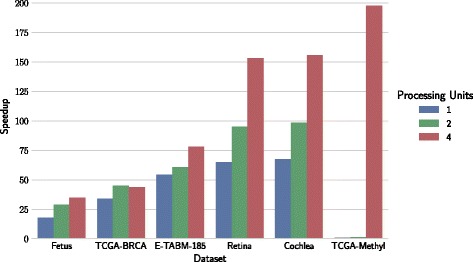
Computational speedups on multi-GPU architectures. Speedup on 1-, 2- and 4-GPU devices is compared to a single-processor configuration. Datasets are ordered from smallest to largest based on the number of non-zero values

Figures 4 and 5 show speedups achieved on multi-processing and multi-GPU architectures, respectively, for each of six considered biomedical datasets. Runtime performance was tested on architectures with one, two or four processing units. Data matrices were partitioned according to block configurations in Table 2.

**Table 2:** Block configurations used in experiments where we tested architectures with two or four processing units (PUs)

Dataset	Data type	2 PUs	4 PUs
Fetus	sparse	2×1	2×2
TCGA-BRCA	dense	1×2	1×4
E-TABM-185	dense	2×1	4×1
Retina	dense	2×1	2×2
Cochlea	dense	2×1	2×2
TCGA-Methyl	dense	1×2 (CPU only)	1×4

**Fig. 6:**
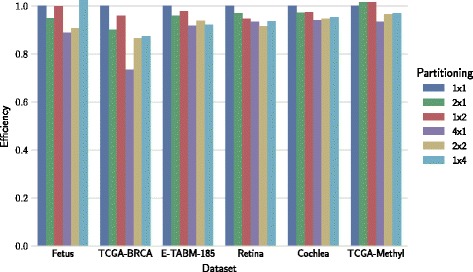
Efficiency of multi-processor architectures for different block configurations. Efficiency is represented by the fraction of linear speedup. The number of parallel processes is equal to the number of partitions

**Fig. 7:**
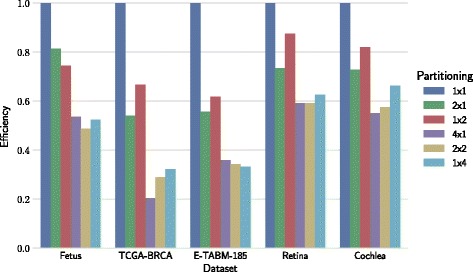
Efficiency of multi-GPU architectures for different block configurations. Efficiency is represented by the fraction of linear speedup. The number of GPU devices is equal to the number of matrix blocks

Efficiency of parallel implementation depends on dataset shape and on chosen block configuration. Measurements illustrating this dependency are shown in Fig. 6 for multi-processor architectures and in Fig. 7 for multi-GPU architectures.

One bottleneck of GPU-based architectures is communication overhead that occurs when copying data between GPU boards. This overhead was also observed in our experiments. For example, up to 50% of time needed to factorize TCGA-BRCA dataset in a 4-GPU environment was spent for communication. On larger datasets, however, this overhead was less pronounced. A detailed analysis is provided in Additional file 1: Figures S6 and S8. The communication overhead on multi-processor architectures is negligible as shown in the Additional file 1: Figures S5 and S7.

**Fig. 8:**
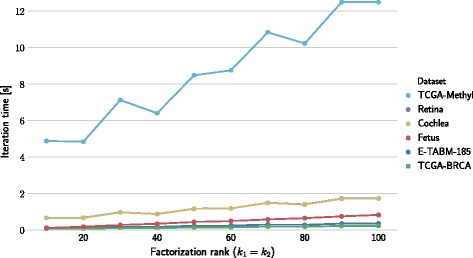
Iteration time depending on factorization rank in a 4-processor environment. The average runtime of one iteration is plotted against factorization rank. Factorization rank is represented by parameters *k*_1_=*k*_2_

**Fig. 9:**
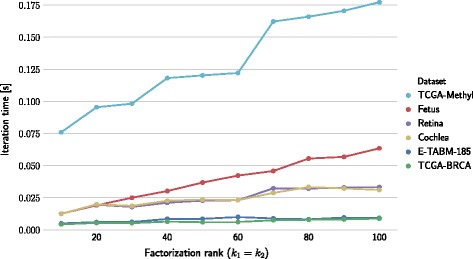
Iteration time depending on factorization rank in a 4-GPU environment. The average runtime of one iteration is plotted against factorization rank. Factorization rank is represented by parameters *k*_1_=*k*_2_

We also studied algorithm scalability with respect to factorization rank. Figure 9 shows runtime of one iteration as a function of factorization rank value on a four-GPU architecture using a 2×2-block configuration. Figure 8 shows the results on a four-processor architecture.

Using matrix partitioning approach presented in Algorithm 1, we can increase speedup on sparse datasets that have imbalanced distribution of nonzero elements. The approach adapts matrix block size based on the number of nonzero elements. In Additional file 1: Figure S11 we show factorization speedup attributed to the adaptive nature of Algorithm 1, and compared to non-adaptive partitioning of data matrix into equally sized blocks. We observe a speedup of up to 1.4-times on multi-processor architecture, and up to 1.2-times on multi-GPU architecture.

## Discussion

### Speedup

Speedup on GPU-architectures is substantial, and pronounced with the dataset size and number of GPUs. For example, factorization on a retina dataset was 150-times faster than that on a single processor. Datasets in Fig. 5 are ordered by their number of nonzero elements, and we can observe a steady increase in speedup. Similar trends can also be observed on multi-processor architectures (Fig. 4), but the speedups are substantially lower than those on the GPUs.

For TCGA-Methyl dataset, the complete data matrix occupies about 19 GBytes of GPU’s memory (Additional file 1: Table S1). With a 12 GBytes of total memory on each GPU, and considering the overhead of libraries and temporary data matrices for inter-GPU communication, the data does not fit to the working memory in 1×1 and 1×2 block configurations. Running the factorization with 1×4 block configurations on 1-GPU or 2-GPU is feasible, but due to insufficient memory to store all necessary blocks in a single GPU requires a transfer of data between main memory and GPUs which severely impacts the runtime and prohibits any speedup. On this large dataset, a configuration with 4-GPUs has sufficient memory and provides for excellent speed-up (Fig. 5). This case also demonstrates that for large datasets the proposed approach requires setups with the adequate number of GPUs that can keep all the data in working GPU memory.

### Efficiency effects of block configuration

Block configuration plays a significant role in minimizing the impact of data transfers and balancing the load across devices (Figs. 6 and 7). Tall datasets (E-TABM-185) favor row-wise partitioning (e.g., 2×1 and 4×1). Wide datasets (TCGA-BRCA, TCGA-Methyl) favor column-wise partitioning (1×2 and 1×4). The two mentioned datasets, E-TABM-185 and TCGA-BRCA, are also those where the effect of the block configuration on efficiency was most pronounced. This observation highlights that suitable block configuration is data dependent, and also indicates that the selection of block configuration can be automated.

The drop in efficiency under a particular choice of block configuration can be explained by increased communication overhead (Additional file 1: Figures S5 and S6). As we increase the number of devices that run in parallel, we need to perform additional data transfers that are not needed on setups with one matrix block. For example, in the case of tall dataset E-MTAB-185 and column-wise partitioning (1×4), over 40% of factorization runtime was spent for transferring data between GPUs. On the other hand, in the case of wide TCGA-BRCA dataset, the lowest efficiency was measured when row-wise partitioning (4×1) was used, because communication cost was the highest.

### Factorization rank

Next, we evaluate the performance of our approach when varying the value of the matrix factorization rank. Factorization rank is a vital parameter of all matrix factorization methods because it determines the number of latent vectors. A larger factorization rank means the inferred latent model has a larger degree of freedom and can thus better approximate the input data matrix [43]. However, increasing factorization rank demands more computational resources and can result in poorer generalization performance [44]. Instead of determining the optimal factorization rank for a given dataset, our goal here is to investigate how the scalability of the proposed block-wise matrix factorization algorithm depends on the value of the factorization rank and on the sparsity of the input data matrix.

Figure 8 shows the iteration time of NMTF as a function of factorization rank on a 4-processor architecture. We can observe that by increasing the factorization rank, the time of iteration increases linearly. For this analysis, both parameters *k*_1_ and *k*_2_, were set with equal values and shown as a single factorization rank parameter. Partitioning was done according to Table 2. When using a single process, the iteration time is proportionally slower according to the speedup shown in Fig. 4.

Figure 9 shows results that correspond to iteration time on 4-GPU architecture. We can see step-wise increases in iteration time, which is a result of the way the multiplication kernel utilizes the physical resources of the GPU [45]. The multiplication on the GPU is done on tiles of data which are processed by several threads in parallel. If the matrix shape is not aligned to the tile size, the border tiles will not make full use of the resources [46].

When comparing the factorization time of a sparse dataset (Fetus) and dense datasets (Retina, Cochlea) of similar size, the benefits of using sparse data structure are substantial. On a GPU, the factorization time on a sparse dataset (Fetus) is slower than on comparable dense datasets (Retina, Cochlea). This is because multiplication with sparse structures requires slower non-sequential memory access [47].

### Interpretation of factorization results

Matrix factorization methods can be used to gain a better understanding of the data and their relationships as the methods identify cluster structures and detect potential new associations. The latent factors learned by NMTF reveal clusters in each of the two dimensions of the input data (matrices **U** and **V**) and encode cluster interactions (matrix **S**). The analysis of the latent factors can then lead to data interpretation, cluster discovery, and to prediction of new interactions.

We here demonstrate that tri-factorization can lead to the reconstruction of biologically meaningful interactions. We have used a DNA methylation dataset (TCGA-Methyl, Table 1) consisting of 10,181 tissue samples from 33 cancer types. Tissue samples are profiled using methylation beta values for 485,577 CpG sites of the DNA. From these, we have considered only the sites that are related to 567 genes with known cancer interactions as listed in the Sanger cancer catalog [48]. Of those, 491 genes were included in our dataset and altogether involved 14,299 methylation sites. The resulting matrix had 10,181 rows and 14,299 columns. We factorized the matrix using factorization ranks *k*_1_=25, *k*_2_=30, which yielded an optimal data compression with respect to the accuracy evaluated on a validation dataset (see Additional file 1: Figure S15).

Additional file 1: Table S2 lists five resulting cluster pairs that relate clusters of genes (from matrix **V**) and clusters of cancer types (from matrix **U**) with highest interaction scores in matrix **S**. First, we note that factorization revealed related cancer types, with, for example, colon, stomach and rectum adenocarcinoma (Additional file 1: Table S2, first row) forming its own group. Also, we found several common Gene Ontology annotations for the clustered genes (Additional file 1: Table S3). Most importantly, we found evidence in published literature for a majority of interactions between genes and cancer types inferred through matrix tri-factorization. For example, *GATA2* was suggested as a prospective indicator for poor prognosis in patients with colorectal cancer [49], and *FAT4* functions as a tumor suppressor for stomach cancer [50]. Other supporting publications are listed in Additional file 1: Table S2.

Transcriptional silencing by DNA methylation plays an important role in the onset of cancer [51, 52]. It is thus encouraging that some of the critical interactions between methylated genes and diseases can be inferred, as demonstrated by this analysis, by non-negative matrix factorization of methylation cancer data alone.

## Conclusion

Non-negative matrix tri-factorization is a successful modeling approach that can reveal hidden patterns in biomedical datasets. Current serial factorization approaches take substantial runtime, particularly for larger datasets. We proposed a block-wise approach to speed up matrix tri-factorization through parallel execution. Experiments show the approach easily scales to very large datasets, and can achieve speedups of up to two orders of magnitude on current GPU-based architectures. We anticipate the proposed approach will be important for data integration, where matrix tri-factorization on large collections of data matrices [53] has already been proven superior and where execution times run into days.

## Additional file

Additional file 1 Document with mathematical proofs, results for impact of communication, impact of balancing sparse datasets and results for orthogonal NMTF. (PDF 317 kb)

## Abbreviations

CPU: Central processing unit
GPU: Graphics processing unit
MPI: Message passing interface
NMTF: Non-negative matrix tri-factorization
PU: Processing unit
RNA: Ribonucleic acid
