# Supplementary material for: Scalable non-negative matrix tri-factorization
Source: BioData Min. 2017 Dec 29;10:41. doi: 10.1186/s13040-017-0160-6 (PMC5746986; doi:10.1186/s13040-017-0160-6)
Supplement: Additional file 1 — Document with mathematical proofs, results for impact of communication, impact of balancing sparse datasets and results for orthogonal NMTF. (PDF 317 kb) [file 13040_2017_160_MOESM1_ESM.pdf]

Scalable non-negative matrix tri-factorization: Supplementary material

Andrej Čopar[1], Marinka Žitnik[1,2], and Blaž Zupan[1,3,*]

[1]University of Ljubljana, Faculty of Computer and Information Science, Večna pot 113, Ljubljana, Slovenia
[2]Department of Computer Science, Stanford University, Stanford, CA 94305, USA
[3]Baylor College of Medicine, Houston, TX 77030, USA
[*]blaz.zupan@fri.uni-lj.si

ABSTRACT

We provide further details on performance analysis for our block-wise matrix tri-factorization. In particular, we include analysis of orthogonal matrix tri-factorization that is discussed in our manuscript but whose results, due to conceptual similarity with non-orthogonal factorization were not included in there. We also present the impact of communication overhead on both non-orthogonal and orthogonal NMTF models and show results on the scalability of 4-processor and 4-GPU implementations with regards to factorization rank. Finally, we analyze the speed-up of balanced partitioning.

Contents

1 Equivalence of block-wise and non-block-wise formulation of NMTF **3**
1.1 Updating rule for latent matrix $\mathbf{U}$ . . . 3
1.2 Updating rule for latent matrix $\mathbf{V}$ . . . 4
1.3 Updating rule for latent matrix $\mathbf{S}$ . . . 5
2 Speedup **5**
3 Efficiency **5**
4 Communication overhead **6**
5 Factorization rank **6**
6 Partitioning **6**
7 Memory requirements **6**
8 Co-clustering application on a DNA methylation dataset **7**
References **16**

List of Figures

S1 Speedup of orthogonal NMTF in multi-processor environment. 8
S2 Speedup of orthogonal NMTF in multi-GPU environment. 8
S3 Efficiency of orthogonal NMTF in multi-processor environment. 9
S4 Efficiency of orthogonal NMTF in multi-GPU environment. 9
S5 Communication overhead in multi-processor environment for non-orthogonal NMTF. Impact of communication on efficiency is shown with bright colors. 10
S6 Communication overhead in multi-GPU environment for non-orthogonal NMTF. Impact of communication on efficiency is shown with bright colors. 10
S7 Communication overhead in multi-processor environment for orthogonal NMTF. Impact of communication on efficiency is shown with bright colors. 11
S8 Communication overhead in multi-GPU environment for orthogonal NMTF. Impact of communication on efficiency is shown with bright colors. 11
S9 Iteration time depending on factorization rank in a 4-processor environment for orthogonal NMTF. 12
S10 Iteration time depending on factorization rank in a 4-GPU environment for orthogonal NMTF. 12
S11 Speedup gain with balanced partitioning in multi-processor environment for non-orthogonal NMTF. 13
S12 Speedup gain with balanced partitioning on multi-GPU environment for non-orthogonal NMTF. 13
S13 Speedup gain with balanced partitioning on multi-processor environment for orthogonal NMTF. 14
S14 Speedup gain with balanced partitioning on multi-GPU environment for orthogonal NMTF. 14
S15 Approximation accuracy and factorization rank for a subset of TCGA-methyl dataset. 15

1 Equivalence of block-wise and non-block-wise formulation of NMTF

We show that our block-wise formulation of NMTF is mathematically equivalent to the standard (serial) NMTF, which does not partition a given input data matrix into blocks. That is, the block-wise version of NMTF yields the same latent factors as its non-block-wise counterpart. As a result, the proposed approach for latent factor learning in NMTF has the same predictive power as standard NMTF.

To show this mathematical equivalence we proceed as follows. For each latent matrix **U**, **V**, and **S** we show that its updating rule in the block-wise NMTF is equivalent to the corresponding updating rule in standard NMTF. In particular, we establish a connection between the updating rules in standard NMTF:

$$\mathbf{U} \leftarrow \mathbf{U} \circ \frac{\mathbf{X}\mathbf{V}\mathbf{S}^T}{\mathbf{U}\mathbf{S}\mathbf{V}^T\mathbf{V}\mathbf{S}^T}, \tag{1}$$

$$\mathbf{V} \leftarrow \mathbf{V} \circ \frac{\mathbf{X}^T\mathbf{U}\mathbf{S}}{\mathbf{V}\mathbf{S}^T\mathbf{U}^T\mathbf{U}\mathbf{S}}, \tag{2}$$

$$\mathbf{S} \leftarrow \mathbf{S} \circ \frac{\mathbf{U}^T\mathbf{X}\mathbf{V}}{\mathbf{U}^T\mathbf{U}\mathbf{S}\mathbf{V}^T\mathbf{V}}, \tag{3}$$

and the rules in block-wise NMTF:

$$\mathbf{U}^{(i)} \leftarrow \mathbf{U}^{(i)} \circ \frac{\sum_j \mathbf{X}^{(i,j)}(\mathbf{V}^{(j)}\mathbf{S}^T)}{\mathbf{U}^{(i)}\sum_j(\mathbf{S}((\mathbf{V}^{(j)})^T)(\mathbf{V}^{(j)}\mathbf{S}^T))}, \tag{4}$$

$$\mathbf{V}^{(j)} \leftarrow \mathbf{V}^{(j)} \circ \frac{\sum_i((\mathbf{X}^{(i,j)})^T\mathbf{U}^{(i)})\mathbf{S}}{\mathbf{V}^{(j)}\mathbf{S}^T\sum_i((\mathbf{U}^{(i)})^T\mathbf{U}^{(i)})\mathbf{S}}, \tag{5}$$

$$\mathbf{S} \leftarrow \mathbf{S} \circ \frac{\sum_j\sum_i((\mathbf{U}^{(i)})^T\mathbf{X}^{(i,j)})\mathbf{V}^{(j)}}{\sum_i((\mathbf{U}^{(i)})^T\mathbf{U}^{(i)})\mathbf{S}\sum_j((\mathbf{V}^{(j)})^T\mathbf{V}^{(j)})}. \tag{6}$$

We provide the relevant proofs in Lemmas 1–3.

1.1 Updating rule for latent matrix U

Lemma 1. *We show that the updating rules in Eq. (1) and Eq. (4) are equivalent by showing that every element* u, v *in matrix* **U** *has the same update under both updating rules:*

$$\left(\mathbf{U} \circ \frac{\mathbf{X}\mathbf{V}\mathbf{S}^T}{\mathbf{U}\mathbf{S}\mathbf{V}^T\mathbf{V}\mathbf{S}^T}\right)_{uv} = \left(\mathbf{U}^{(i)} \circ \frac{\sum_j \mathbf{X}^{(i,j)}(\mathbf{V}^{(j)}\mathbf{S}^T)}{\mathbf{U}^{(i)}\sum_j(\mathbf{S}((\mathbf{V}^{(j)})^T)(\mathbf{V}^{(j)}\mathbf{S}^T))}\right)_{uv}. \tag{7}$$

Proof. Note that multiplication ($\circ$) and division ($/$) are element-wise operations and thus the updating rule in Eq. (1) can be written as:

$$U_{uv} \leftarrow U_{uv} \circ \frac{(\mathbf{X}\mathbf{V}\mathbf{S}^T)_{uv}}{(\mathbf{U}\mathbf{S}\mathbf{V}^T\mathbf{V}\mathbf{S}^T)_{uv}}. \tag{8}$$

We therefore only need to show that expressions in numerators and denominators of the updating rules are equivalent. In particular, we need to show that the following holds:

$$(\mathbf{X}\mathbf{V}\mathbf{S}^T)_{uv} = (\sum_j \mathbf{X}^{(i,j)}(\mathbf{V}^{(j)}\mathbf{S}^T))_{uv}, \tag{9}$$

$$(\mathbf{U}\mathbf{S}\mathbf{V}^T\mathbf{V}\mathbf{S}^T)_{uv} = (\mathbf{U}^{(i)}\sum_j(\mathbf{S}((\mathbf{V}^{(j)})^T)(\mathbf{V}^{(j)}\mathbf{S}^T)))_{uv}. \tag{10}$$

Using definition of the matrix product $(AB)_{ij} = \sum_{k=1}^{m} A_{ik}B_{kj}$ and the right-associativity rule of matrix product we get:

$$\sum_{x=1}^{k}(\mathbf{X}\mathbf{V})_{ux}S_{vx} = \sum_{x=1}^{k}(\sum_j \mathbf{X}^{(i,j)}\mathbf{V}^{(j)})_{ux}S_{vx}. \tag{11}$$

Expressions in the left and the right hand side of Eq. (11) are equivalent when the following holds:

$$(\mathbf{X}\mathbf{V})_{ux} = (\sum_j \mathbf{X}^{(i,j)}\mathbf{V}^{(j)})_{ux}, \tag{12}$$

which can be simply shown by writing the product $\mathbf{XV}$ as a sum of element-wise operations:

$$\sum_{y=1}^{m} X_{uy}V_{yx} = \sum_j (\sum_{y=1}^{m_j} \mathbf{X}_{uy}^{(i,j)} \mathbf{V}_{yx}^{(j)}), \tag{13}$$

and observing that u is assigned to the i-th block and that $\sum_j m_j = m$. We use the same approach to show that expressions in denominators of the updating rules are equivalent:

$$(\mathbf{USV}^T\mathbf{VS}^T)_{uv} = (\mathbf{U}^{(i)} \sum_j (\mathbf{S}((\mathbf{V}^{(j)})^T)(\mathbf{V}^{(j)}\mathbf{S}^T)))_{uv}, \tag{14}$$

$$\sum_x U_{ux}(\mathbf{SV}^T\mathbf{VS}^T)_{xv} = \sum_x (\mathbf{U}^{(i)})_{ux}(\mathbf{S}(\sum_j \mathbf{V}^{(j)^T}\mathbf{V}^{(j)})\mathbf{S}^T)_{xv}. \tag{15}$$

Since $\mathbf{V}^T\mathbf{V} = \sum_j \mathbf{V}^{(j)^T}\mathbf{V}^{(j)}$ holds true, we can simplify the expression in Eq. (15) to: $U_{ux} = (\mathbf{U}^{(i)})_{ux}$. Finally, we conclude that Eq. (14) holds true for every u, x, i, where $U_{ux} \in \mathbf{U}^{(i)}$. This concludes our proof. □

1.2 Updating rule for latent matrix V

Lemma 2. *We show that the updating rules in Eq. (2) and Eq. (5) are equivalent by showing that every element u, v in matrix $\mathbf{V}$ has the same update under both updating rules:*

$$\left(\mathbf{V} \circ \frac{\mathbf{X}^T\mathbf{US}}{\mathbf{VS}^T\mathbf{U}^T\mathbf{US}}\right)_{uv} = \left(\mathbf{V}^{(j)} \circ \frac{\sum_i ((\mathbf{X}^{(i,j)})^T\mathbf{U}^{(i)})\mathbf{S}}{\mathbf{V}^{(j)}\mathbf{S}^T \sum_i ((\mathbf{U}^{(i)})^T\mathbf{U}^{(i)})\mathbf{S}}\right)_{uv}. \tag{16}$$

Proof. Note that multiplication ($\circ$) and division ($/$) are element-wise operations and thus the updating rule in Eq. (2) can be written as:

$$V_{uv} \leftarrow V_{uv} \circ \frac{(\mathbf{X}^T\mathbf{US})_{uv}}{(\mathbf{VS}^T\mathbf{U}^T\mathbf{US})_{uv}}. \tag{17}$$

We therefore only need to show that expressions in numerators and denominators of the updating rules are equivalent. In particular, we need to show that the following holds:

$$(\mathbf{X}^T\mathbf{US})_{uv} = (\sum_i ((\mathbf{X}^{(i,j)})^T\mathbf{U}^{(i)})\mathbf{S})_{uv}, \tag{18}$$

$$(\mathbf{VS}^T\mathbf{U}^T\mathbf{US})_{uv} = (\mathbf{V}^{(j)}\mathbf{S}^T \sum_i ((\mathbf{U}^{(i)})^T\mathbf{U}^{(i)})\mathbf{S})_{uv}. \tag{19}$$

Using definition of the matrix product $(AB)_{ij} = \sum_{k=1}^m A_{ik}B_{kj}$ and the right-associativity rule of matrix product we get:

$$\sum_{x=1}^{k} (\mathbf{X^TU})_{ux}S_{xv} = \sum_{x=1}^{k} (\sum_i \mathbf{X}^{(i,j)^T}\mathbf{U}^{(i)})_{ux}S_{xv}. \tag{20}$$

Expressions in the left and the right hand side of Eq. (20) are equivalent when the following holds:

$$(\mathbf{X}^T\mathbf{U})_{ux} = (\sum_i (\mathbf{X}^{(i,j)^T}\mathbf{U}^{(i)})_{ux}, \tag{21}$$

which can be simply shown by writing the product $\mathbf{X}^T\mathbf{U}$ as a sum of element-wise operations:

$$\sum_{y=1}^{n} X_{yu}U_{yx} = \sum_i \sum_{y=1}^{n_j} (\mathbf{X}^{(i,j)})_{yu}(\mathbf{U}^{(i)})_{yx}, \tag{22}$$

and observing that u is assigned to the i-th block and that $\sum_j m_j = m$. We use the same approach to show that expressions in denominators of the updating rules are equivalent:

$$(\mathbf{VS}^T\mathbf{U}^T\mathbf{US})_{uv} = (\mathbf{V}^{(j)}\mathbf{S}^T \sum_i ((\mathbf{U}^{(i)})^T\mathbf{U}^{(i)})\mathbf{S})_{uv}, \tag{23}$$

$$\sum_x V_{ux}(\mathbf{S}^T\mathbf{U}^T\mathbf{US})_{xv} = \sum_x \mathbf{V}^{(j)})_{ux}(\mathbf{S}^T \sum_i ((\mathbf{U}^{(i)})^T\mathbf{U}^{(i)})\mathbf{S})_{xv}. \tag{24}$$

Since $\mathbf{U}^T\mathbf{U} = \sum_i \mathbf{U}^{(i)^T}\mathbf{U}^{(i)}$ holds true, we can simplify the expression in Eq. (24) to: $V_{ux} = (\mathbf{V}^{(j)})_{ux}$. Finally, we conclude that Eq. (23) holds true for every u, x, j, where $V_{ux} \in \mathbf{V}^{(j)}$. This concludes our proof. □

1.3 Updating rule for latent matrix $\mathbf{S}$

Lemma 3. *We show that the updating rules in Eq. (3) and Eq. (6) are equivalent by showing that every element u, v in matrix $\mathbf{S}$ has the same update under both updating rules:*

$$\left(\mathbf{S}\circ\frac{\mathbf{U}^T\mathbf{X}\mathbf{V}}{\mathbf{U}^T\mathbf{U}\mathbf{S}\mathbf{V}^T\mathbf{V}}\right)_{uv} = \left(\mathbf{S}\circ\frac{\sum_j\sum_i((\mathbf{U}^{(i)})^T\mathbf{X}^{(i,j)})\mathbf{V}^{(j)}}{\sum_i((\mathbf{U}^{(i)})^T\mathbf{U}^{(i)})\mathbf{S}\sum_j((\mathbf{V}^{(j)})^T\mathbf{V}^{(j)})}\right)_{uv}. \tag{25}$$

Proof. Note that multiplication ($\circ$) and division ($/$) are element-wise operations and thus the updating rule in Eq. (3) can be written as:

$$S_{uv} \leftarrow S_{uv}\circ\frac{(\mathbf{U}^T\mathbf{X}\mathbf{V})_{uv}}{(\mathbf{U}^T\mathbf{U}\mathbf{S}\mathbf{V}^T\mathbf{V})_{uv}}. \tag{26}$$

We therefore only need to show that expressions in numerators and denominators of the updating rules are equivalent. In particular, we need to show that the following holds:

$$(\mathbf{U}^T\mathbf{X}\mathbf{V})_{uv} = (\sum_j\sum_i((\mathbf{U}^{(i)})^T\mathbf{X}^{(i,j)})\mathbf{V}^{(j)})_{uv}, \tag{27}$$

$$(\mathbf{U}^T\mathbf{U}\mathbf{S}\mathbf{V}^T\mathbf{V})_{uv} = (\sum_i((\mathbf{U}^{(i)})^T\mathbf{U}^{(i)})\mathbf{S}\sum_j((\mathbf{V}^{(j)})^T\mathbf{V}^{(j)}))_{uv}. \tag{28}$$

Using definition of the matrix product $(AB)_{ij} = \sum_{k=1}^m A_{ik}B_{kj}$ and the right-associativity rule of matrix product we get:

$$\sum_{x=1}^k U_{xu}(\mathbf{X}\mathbf{V})_{xv} = \sum_{x=1}^k(\sum_j\sum_i(U^{(i)})_{xu}(\mathbf{X}^{(i,j)}\mathbf{V}^{(j)})_{xv}). \tag{29}$$

Expressions in the left and the right hand side of Eq. (29) are equivalent when U_{ux} is a member of $\mathbf{U}^{(i)}$ and the following holds:

$$(\mathbf{X}\mathbf{V})_{xv} = \sum_j\sum_i(\mathbf{X}^{(i,j)}\mathbf{V}^{(j)})_{xv}, \tag{30}$$

which can be written as a sum of element-wise operations:

$$\sum_{y=1}^m X_{xy}V_{yv} = \sum_j\sum_i(\sum_{y=1}^{m_j}\mathbf{X}_{xy}^{(i,j)}\mathbf{V}_{yv}^{(j)}). \tag{31}$$

Eq. (31) holds true for every x, y, i, j, where $X_{xy}\in\mathbf{X}^{(i,j)}$ and for every y, v, j, where $\mathbf{V}_{yv}\in V^{(j)}$. We use the same approach to show that expressions in denominators of the updating rules are equivalent:

$$(\mathbf{U}^T\mathbf{U}\mathbf{S}\mathbf{V}^T\mathbf{V})_{uv} = (\sum_i((\mathbf{U}^{(i)})^T\mathbf{U}^{(i)})\mathbf{S}\sum_j((\mathbf{V}^{(j)})^T\mathbf{V}^{(j)}))_{uv}. \tag{32}$$

Since $\mathbf{U}^T\mathbf{U} = \sum_i \mathbf{U}^{(i)^T}\mathbf{U}^{(i)}$ and $\mathbf{V}^T\mathbf{V} = \sum_j \mathbf{V}^{(j)^T}\mathbf{V}^{(j)}$ hold true, we can simplify the expression on the right hand side of Eq. (32) to $(\mathbf{U}^T\mathbf{U}\mathbf{S}\mathbf{V}^T\mathbf{V})_{uv}$, which is equivalent to the left hand side. This concludes our proof.

□

2 Speedup

Figure S1 shows the speedup achieved with parallelization in a multi-processor environment. Figure S2 shows the speedup of orthogonal NMTF in 1-,2- and 4-GPU environments compared to a serial approach with a single CPU process. Note that the speedup in almost identical to non-orthogonal NMTF.

3 Efficiency

Here, we show the effect of different block configurations on orthogonal NMTF. Figure S3 shows the impact on the efficiency of multi-processor implementations, where the number of processors is the same as the number of partitions. On Figure S4 we show the efficiency of orthogonal NMTF on multi-GPU configurations. On both figures, the number of processing units is the same as the number of blocks. For example, 2x1 block configuration runs on 2 GPUs and 2x2 configuration runs on 4 GPUs simultaneously.

4 Communication overhead

The main reason for the drop in efficiency of multi-GPU environments is communication overhead. The comparison of efficiency (darker colors) compared to the efficiency that would be achieved without data transfers (bright colors) for non-orthogonal NMTF is shown on Figures S5 for multi-processor and S6 for multi-GPU architectures. We can see that the communication overhead has almost no impact on multi-processor architecture since the different processor cores share the same global memory. However, the impact of communication on multi-GPU architectures is substantial, because it requires transferring the data between GPU devices.

Similarly, the communication overhead for orthogonal NMTF is shown on Figure S7 for multi-processor and S8 for multi-GPU architecture. Larger datasets that are shown towards the right side require more computation, which reduces the impact of communication overhead on efficiency.

5 Factorization rank

On Figure S9 we show the impact of factorization rank on runtime of 4-processor 2x2 configuration for orthogonal NMTF. Figure S10 shows runtime of 4-GPU implementations. In the multi-processor environment, we observe an almost linear increase in runtime depending on factorization rank. In the multi-GPU environment, we observe better scalability when using dense matrix representation, where runtime on a sparse dataset (shown with blue line) appears to increase more rapidly. From these figures, we can conclude that the GPUs are better optimized for dealing with dense matrices and we can expect greater speedup over multi-processor environment when dealing with higher factorization rank or larger input dataset.

6 Partitioning

On sparse datasets, the partitioning can be done either by splitting the data into blocks of equal size or with regards to the number of nonzero elements. On sparse dataset, the computational complexity depends on the number of nonzero elements. Balancing the number of non-zero elements in each block better distributes the workload across different devices.

With the proposed balanced partitioning algorithm we can increase the speedup up to 1.4 times on multi-processor architectures, as shown in Figures S11 and S13 for non-orthogonal and orthogonal NMTF respectively. Similarly, we can observe speedup of up to 1.2 times on multi-GPU architectures, shown on Figures S12 for non-orthogonal and S14 orthogonal NMTF model. These observations suggest that the GPU architecture is more robust to the distribution of non-zero elements in the data.

7 Memory requirements

Table 1 shows an example of CPU and GPU usage on setup with four processing units. The memory requirements are proportional to the data size, in particular, each value needs 4 bytes of memory; hence the total memory usage can be approximated as four times the size of the data. Note that in addition to the reported numbers, GPU implementation requires approximately 300MB per GPU for software libraries. When running on multiple GPUs, data is distributed, and requirements per device are proportionally smaller.

Table 1. Total CPU and GPU memory usage in megabytes (MB).

Dataset	4-CPU	4-GPU
Fetus	290	505
TCGA-BRCA	298	300
E-TABM-185	509	515
Retina	2595	2566
Cochlea	2595	2566
TCGA-Methyl	18937	18949

8 Co-clustering application on a DNA methylation dataset

We illustrate the interpretation of NMTF results on the factorization of DNA methylation data set, where input data consist of 10,181 tissue samples from any of the 33 cancer types. Tissue samples are profiled with methylation scores at 485,577 CG sites of the DNA. From these, we have considered only the sites that are related to 567 genes with known cancer interactions as listed in the Sanger cancer catalog[1]. Of these, 491 genes were included in our dataset, which collectively encompasses 14,299 methylation sites. The resulting matrix of the size $10,181 \times 14,299$ was factorized with factorization ranks $k_1 = 25$, $k_2 = 30$, which yielded optimal data compression with respect to retained accuracy when tested on validation data set (see Figure S15). Five resulting cluster pairs that relate clusters of genes (from matrix $\mathbf{V}$) and clusters of cancers (from matrix $\mathbf{U}$) with highest interaction scores (from matrix $\mathbf{S}$) are listed in Table 2.

We link each of the interactions with supporting evidence that describe the interactions between a gene with one of the three representative cancer types. In particular, gene fusion transcripts of *RSPO3* are associated with colorectal cancer [1], *GATA2* was suggested as a prospective indicator for poor prognosis in patients with colorectal cancer [2] and *FAT4* functions as a tumor suppressor for stomach cancer [3]. *CXCR4* plays an important role in glioblastoma cells [4], where *WWTR1* has been reported to be overexpressed in several human cancers including lung cancer [5]. Changes in the expression of the *WT1* gene are associated with several forms of cancer, including prostate cancer [6] and breast cancer [7]. HOXA9 methylation patterns were shown to be a prognostic marker in bladder cancer patients [8]. The common Gene Ontologies for the three representatives of each gene cluster are shown Table 3. In the first group, *RSPO3* does not share common Gene Ontology with the other two genes *GATA2* and *FAT4*.

Table 2. Five strongest interactions between gene and cancer type clusters.

Gene cluster	Cancer type cluster
RSPO3 [1], GATA2 [2], FAT4 [3]	Colon Adenocarcinoma, Stomach Adenocarcinoma, Rectum Adenocarcinoma
CXCR4 [4], BIRC3, WWTR1 [5]	Brain Lower Grade Glioma, Glioblastoma Multiforme, Lung Adenocarcinoma
CCND2, FEV, WT1 [6,7]	Prostate Adenocarcinoma, Breast Invasive Carcinoma, Adrenocortical Carcinoma
CCND2, FEV, WT1 [6,7]	Breast Invasive Carcinoma, Prostate Adenocarcinoma, Uterine Corpus Endometrial Carcinoma
HLA-A, HOXA9 [8], VHL	Bladder Urothelial Carcinoma, Lung Squamous Cell Carcinoma, Cervical Squamous Cell Carcinoma

Table 3. Common GO terms for each gene group in Table 2.

Gene cluster	Common GO term
GATA2, FAT4	protein binding
CXCR4, BIRC3, WWTR1	cytoplasm, protein binding
CCND2, FEV, WT1	nucleus
HLA-A, HOXA9, VHL	protein binding

[1] http://cancer.sanger.ac.uk/census/

Figure S1. Speedup of orthogonal NMTF in multi-processor environment.

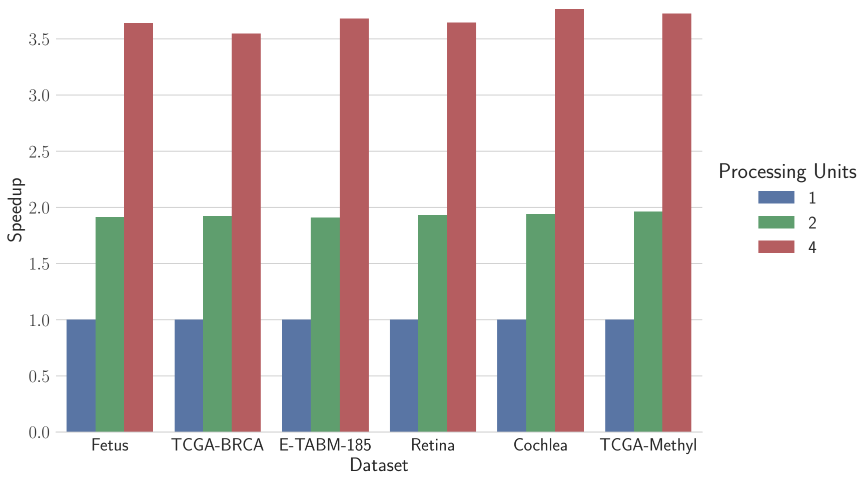

Figure S2. Speedup of orthogonal NMTF in multi-GPU environment.

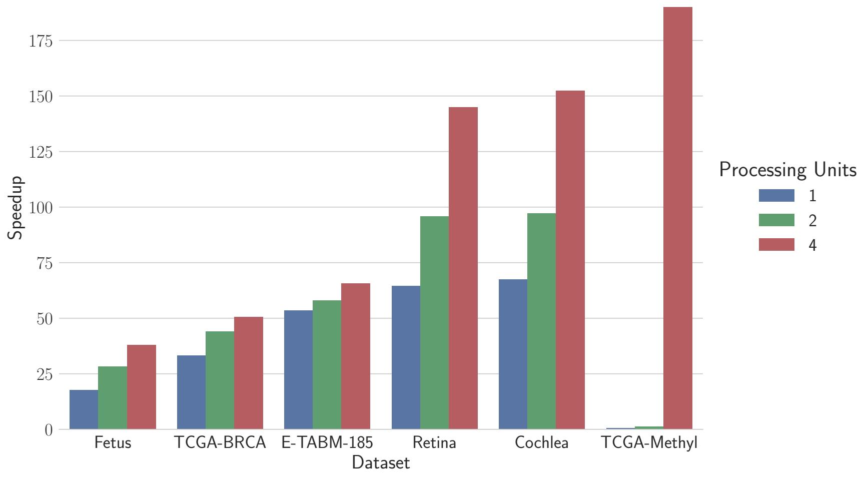

Figure S3. Efficiency of orthogonal NMTF in multi-processor environment.

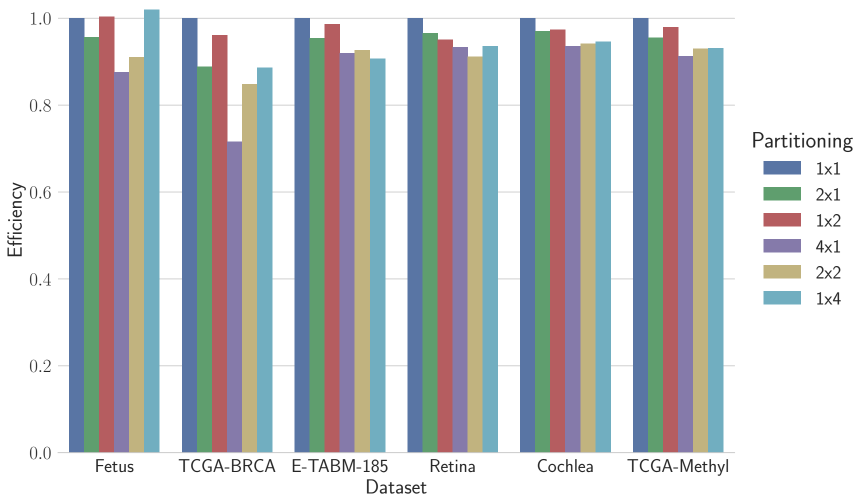

Figure S4. Efficiency of orthogonal NMTF in multi-GPU environment.

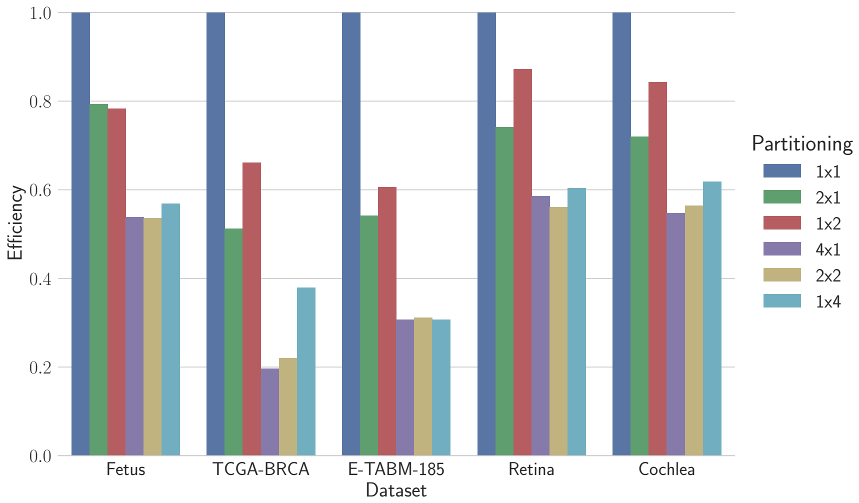

Figure S5. Communication overhead in multi-processor environment for non-orthogonal NMTF. Impact of communication on efficiency is shown with bright colors.

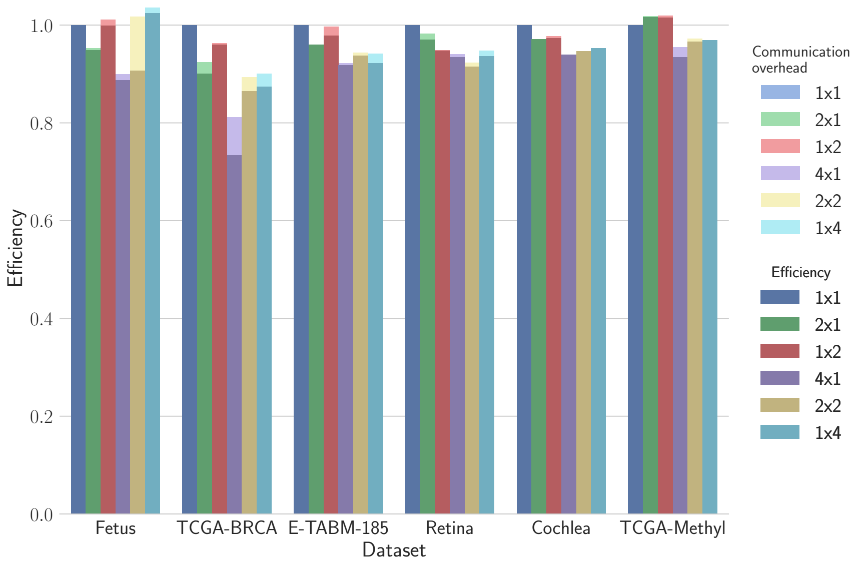

Figure S6. Communication overhead in multi-GPU environment for non-orthogonal NMTF. Impact of communication on efficiency is shown with bright colors.

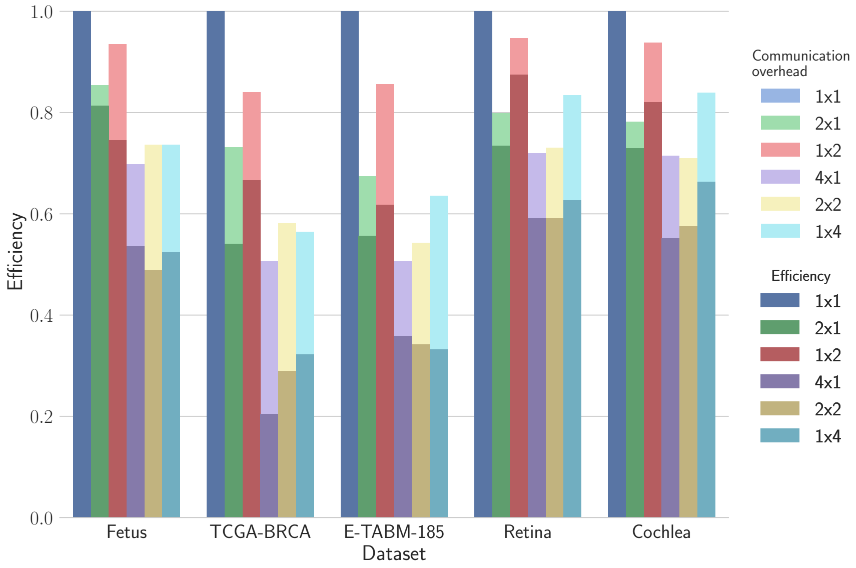

Figure S7. Communication overhead in multi-processor environment for orthogonal NMTF. Impact of communication on efficiency is shown with bright colors.

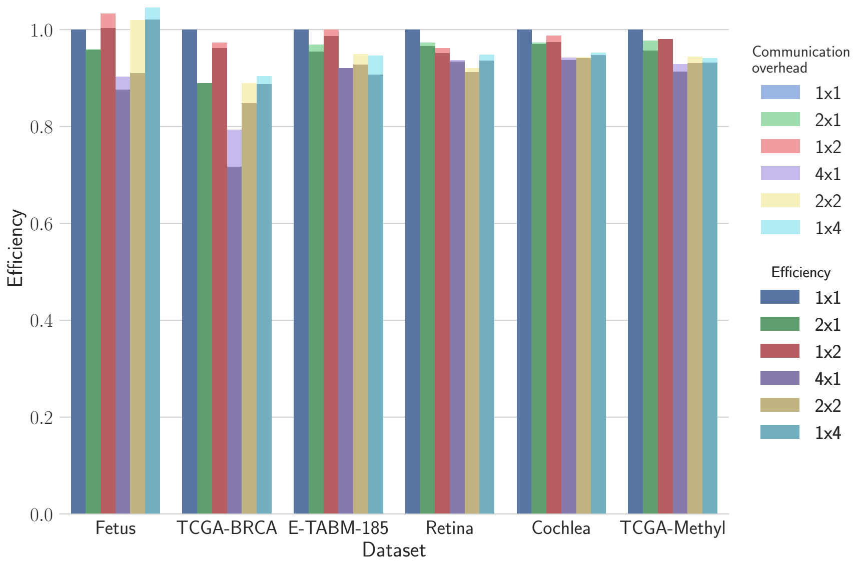

Figure S8. Communication overhead in multi-GPU environment for orthogonal NMTF. Impact of communication on efficiency is shown with bright colors.

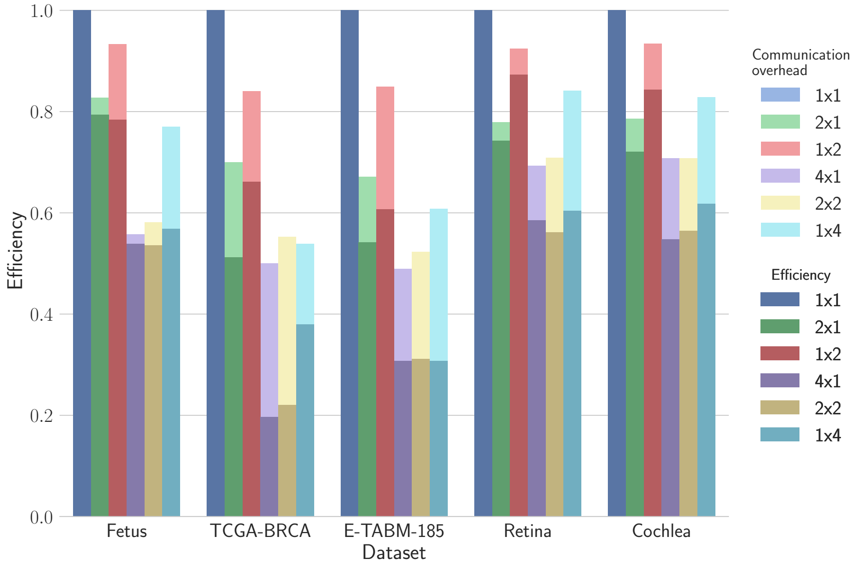

Figure S9. Iteration time depending on factorization rank in a 4-processor environment for orthogonal NMTF.

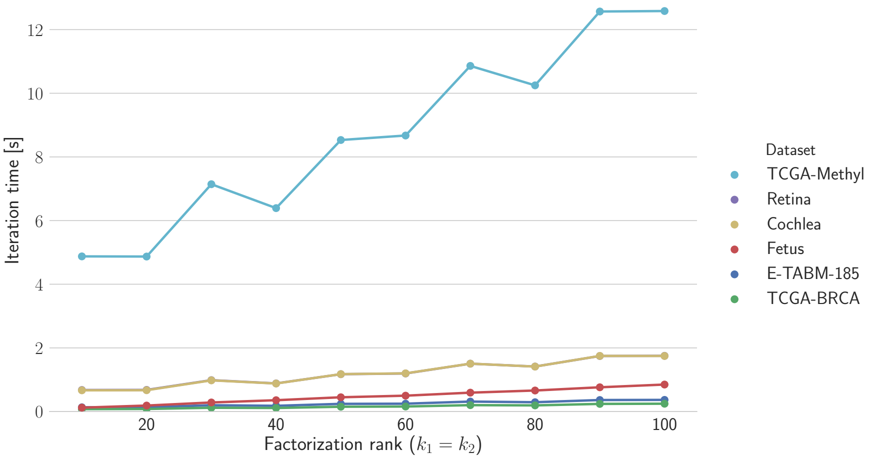

Figure S10. Iteration time depending on factorization rank in a 4-GPU environment for orthogonal NMTF.

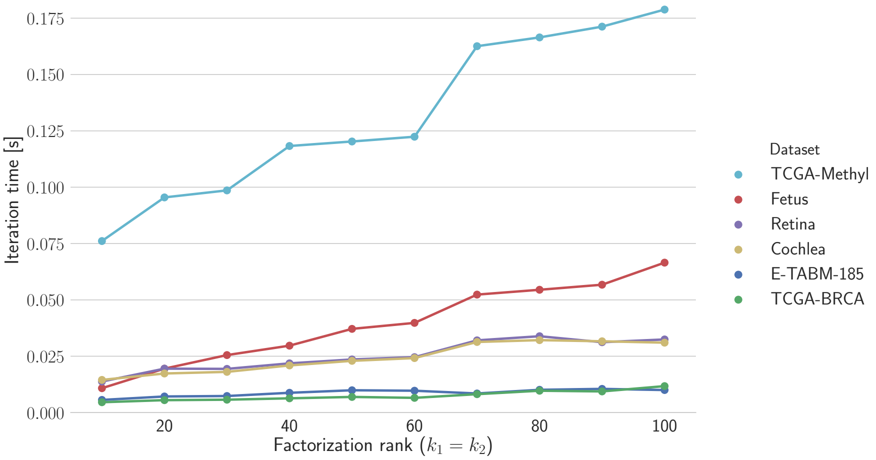

Figure S11. Speedup gain with balanced partitioning in multi-processor environment for non-orthogonal NMTF.

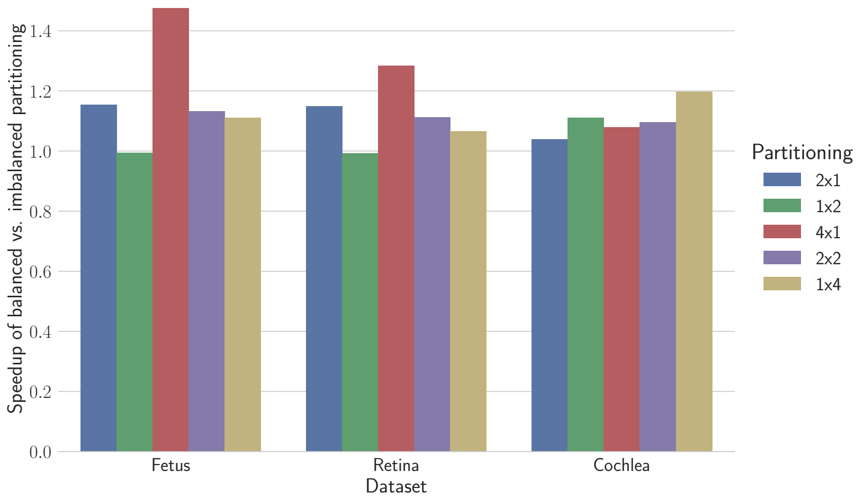

Figure S12. Speedup gain with balanced partitioning on multi-GPU environment for non-orthogonal NMTF.

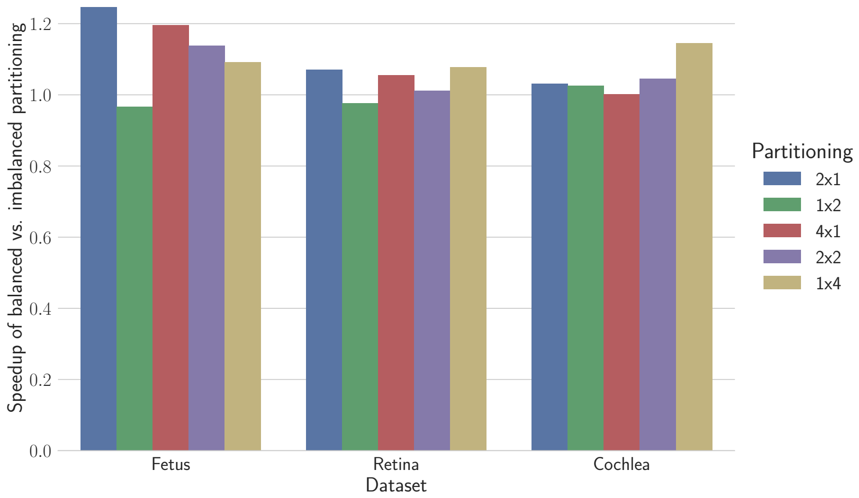

Figure S13. Speedup gain with balanced partitioning on multi-processor environment for orthogonal NMTF.

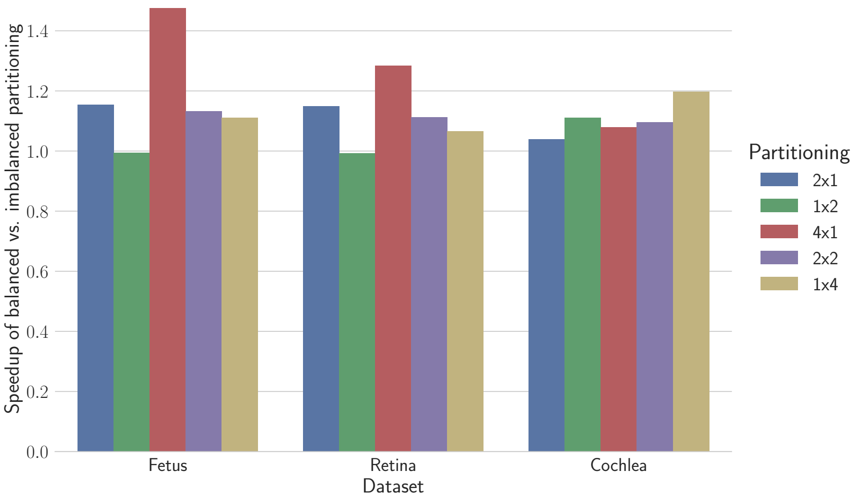

Figure S14. Speedup gain with balanced partitioning on multi-GPU environment for orthogonal NMTF.

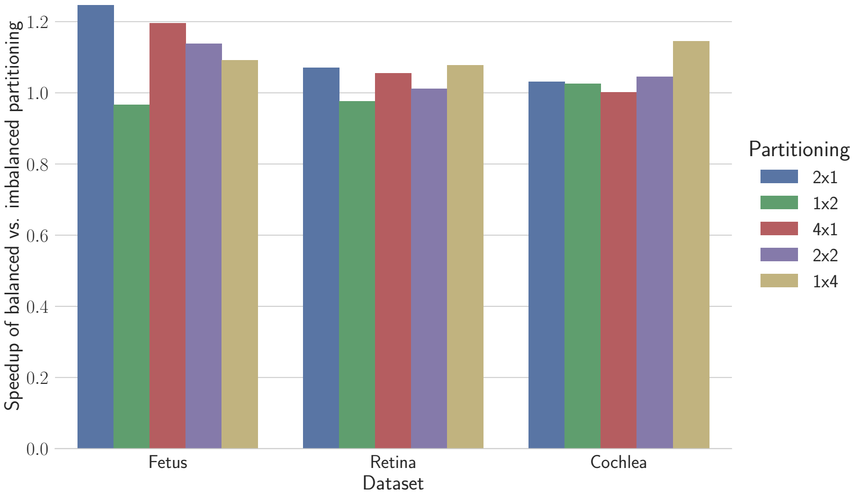

Figure S15. Approximation accuracy and factorization rank for a subset of TCGA-methyl dataset.

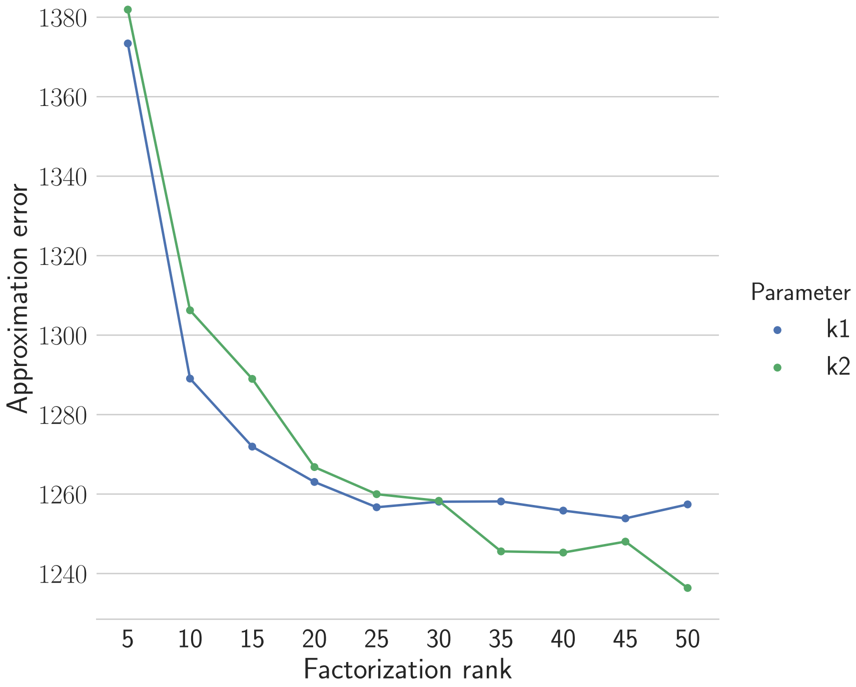

References

1. Seshagiri, S., Stawiski, E.W., Durinck, S., Modrusan, Z., Storm, E.E., Conboy, C.B., Chaudhuri, S., Guan, Y., Janakiraman, V., Jaiswal, B.S., *et al.*: Recurrent R-spondin fusions in colon cancer. Nature **488**(7413), 660 (2012)

2. Xu, K., Wang, J., Gao, J., Di, J., Jiang, B., Chen, L., Wang, Z., Wang, A., Wu, F., Wu, W., *et al.*: GATA binding protein 2 overexpression is associated with poor prognosis in KRAS mutant colorectal cancer. Oncology reports **36**(3), 1672–1678 (2016)

3. Cai, J., Feng, D., Hu, L., Chen, H., Yang, G., Cai, Q., Gao, C., Wei, D.: FAT4 functions as a tumour suppressor in gastric cancer by modulating wnt/β-catenin signalling. British Journal of Cancer **113**(12), 1720 (2015)

4. Ehtesham, M., Mapara, K.Y., Stevenson, C.B., Thompson, R.C.: CXCR4 mediates the proliferation of glioblastoma progenitor cells. Cancer Letters **274**(2), 305–312 (2009)

5. Wang, L., Chen, Z., Wang, Y., Chang, D., Su, L., Guo, Y., Liu, C.: WWTR1 promotes cell proliferation and inhibits apoptosis through cyclin a and ctgf regulation in non-small cell lung cancer. Tumor Biology **35**(1), 463–468 (2014)

6. Devilard, E., Bladou, F., Ramuz, O., Karsenty, G., Dalès, J.-P., Gravis, G., Nguyen, C., Bertucci, F., Xerri, L., Birnbaum, D.: FGFR1 and WT1 are markers of human prostate cancer progression. BMC cancer **6**(1), 272 (2006)

7. Miyoshi, Y., Ando, A., Egawa, C., Taguchi, T., Tamaki, Y., Tamaki, H., Sugiyama, H., Noguchi, S.: High expression of wilms' tumor suppressor gene predicts poor prognosis in breast cancer patients. Clinical Cancer Research **8**(5), 1167–1171 (2002)

8. Kim, Y.-J., Yoon, H.-Y., Kim, J.S., Kang, H.W., Min, B.-D., Kim, S.-K., Ha, Y.-S., Kim, I.Y., Ryu, K.H., Lee, S.-C., *et al.*: HOXA9, ISL1 and ALDH1A3 methylation patterns as prognostic markers for nonmuscle invasive bladder cancer: Array-based DNA methylation and expression profiling. International Journal of Cancer **133**(5), 1135–1142 (2013)
